# Presentiments

**Published:** 1886-08

**Authors:** 


### HALL'S JOURNAL OF HEALTH

VOL. 33. AUGUST, 1886. No. 8

### *SPECIAL NOTICE.*

The business headquarters of HALL'S JOURNAL OF HEALTH have been removed to new and commodious offices at 206 Broadway, Evening Post Building, entrance Room 11, one of the most central locations in the city of New York, to which all communications and "exchanges" should be hereafter addressed, and where all local business relating to the JOURNAL will be transacted.

JOURNAL OF HEALTH PUBLISHING CO.,

206 Broadway.

### *PRESENTIMENTS.*

In the July number of the JOURNAL, we made allusion to its changed attitude towards a class of phenomena, a belief in which it formerly ascribed to a weak and disordered state of the mental faculties.

There is nothing quite so cheap as ridicule in the treatment of subjects which by any other method involve study, investigation and reflection. To throw them off with a jest, is an easy way to get rid of all serious enquiry into their merits, but unfortunately this sort of treatment is never satisfactory to the student, in search of truth, holding it supreme over all else:

> "Slave to no sect, who takes no private road,
> But looks through nature up to nature's God."

In this free thinking age, when so many brilliant intellects are enrolled upon the side of the materialist, who assigns to man a purely animal existence from whose grosser standpoint all the achievements of his superior mentality count for nothing in the great hereafter, it is not surprising that the evidences of his dual nature, should be met with incredulity and denial, for it is one of the weaknesses of the specialist in science, and the unwisely and *incurably learned*, to try everything by their own usually narrow and incomplete standard. But the fact of presentiments is too well-established by incontrovertible proof to be all doubtful in the estimation of any fair minded investigator. There is scarcely a family which has not among its unwritten annals, at least a tradition of some strange premonition or fore-warning of that which was yet to happen, and which subsequent events verified, even as foreshadowed, oftentimes in a manner as unsubstantial as a dream. Time and time again have lives and property been saved from destruction by a timely regard for these interested warnings.

In the middle of the 18th century, Augustine Calmet, a celebrated French liberal scholar and author published a volume of remarkable occurrences, usually assigned to the supernatural, which passed through several editions. A century later, this work was reproduced in English by the Rev. Henry Christmas, M.A.; F.R.S; F.S.A., etc., who in the course of his elaborate introduction writes, "Calmet was a man of naturally cool, calm judgment, possessed of singular learning, and was pious and truthful." Among his principal works were a commentary on the Old and New Testament, and a History of the Bible, both characterized by great learning, and his life was largely devoted to giving instruction to the embryo priesthood upon biblical themes. But when it came to a consideration of the merits of the carefully compiled volume in question, the reverend English translator, true to the prejudices of his priestly office, made free to confess that while he accepted literally, all the marvels related in the New Testament; as to those collected by Calmet, he believed not one of them.

Nevertheless, he concedes that works of this class "are at no time to be regarded as merely subjects of amusement; they have their philosophical value; they have a still greater historical value; and they show how far even upright minds may be warped by imperfect education and slavish defference to authority."

Could it have occurred to this Reverend Commentator, how accurately his words describe the condition of his own mind, and that of thousands of others of trained and particular schools of thought, whose votaries blindly accept their teachings and reject all opposite views as unworthy of serious consideration?

Passing from the volume of Calmet to the more recent works of Mrs. Crowe and Robert Dale Owen, we find them to abound in well anthenticated instances of presentiment, which are doubtless familiar to a portion at least, of our readers.

In commenting upon the receptive faculties of the mind, Mrs. Crowe remarks: "It has been the opinion of many philosophers, both ancient and modern, that in the original state of man, as he came forth from the hands of his Creator, that knowledge which is now acquired by pains and labor, was intuitive. His material body was given him for the purpose of placing him in relation with the material world, and his sensuous organs for the perception of material objects, but his soul was a mirror of the universe, in which everything was reflected, and, probably, is so still, but that the spirit is no longer in a condition to perceive it."

Mrs. Crowe classifies the various phazes of presentiment under the following heads: Allegorical dreams, presentiments, and warnings, giving the particulars of many and various cases of forewarnings of future events in such minute detail as to leave no reasonable doubt of their authenticity.

The later carefully compiled works of Mr. Owen, whose scholarship and proberty none will question, abound in like indisputable instances of the forecasting of future events in dreams, in visions and other extraordinary ways, none the less authentic for being modern and verified by living witnesses.

After informing his readers of the long, patient and laborious course of study and investigation pursued by him, Mr. Owen says: "Gradually, I became convinced that what by many have been regarded as new and unexampled phenomena, are but modern phases of what has ever existed."

In treating of a subject so replete with evidences of individual forewarning it certainly is not out of place to give the particulars of one or two recent occurrences, in the expectation that they will be accepted as true, even by the skeptically minded, under the assurance which we are able to make of their actuality.

It is only a few months ago that a young student residing in Brooklyn, N. Y., was called upon to mourn the transition to the other life of a beloved teacher, and notwithstanding the disparity of age, no less a friend and companion. Not long subsequent to this event, the young student mentioned to some of his intimates that he had seen and conversed with his deceased friend, who had imparted to him the information that on a day named, then some months ahead, he would join him in his new state of existence. All this naturally enough, was looked upon as a meloncholy delusion, and every means were resorted to to turn the young gentleman's thoughts into livelier and happier channels; but the meeting with his whilom teacher, and the conversation and prediction as stated, were insisted upon with a seriousness which occasioned no little apprehension on the part of those who held interested relations with him. As the days passed on the student in question mingled freely with his accustomed associates, sharing in their diversions with no apparent abatement of interest or enjoyment. Only an evening before the period named as his last on earth he was present at a social gathering of both sexes, and none were gayer and happier than he. The only remark made by him in allusion to it was this question to one of the assembled company. "If I send for you to-morrow, will you come to me;" and ere the sun had gone down on that fateful day, his spirit had left its earthly tenement, let us hope, to join his friend and teacher in a sphere of action, no less useful and far more satisfactory than this.

A gentleman of our acquaintance, who for a number of years has interested himself in ferreting out the facts of similar occurrences, addressed a letter to the father of the deceased, from whom he received in reply an account in detail substantially as we give it here.

A no less remarkable instance of presentiment of a different order was lately reported from the South, the truth of which our friend verified in like manner.

A physician saw in a dream the cruel death of a brother, with all the details of a red-handed murder. So vivid was the picture, that he lost no time in journeying to the place thus designated, and though strange to the neighborhood, the street, and the houses, the identical location of the tragedy were as familiar to his eye as the tresured scenes of boyhood, and that which the world in general would have dismissed from mind as "only a dream," proved to be a sad and savage reality. We might multiply the narration of similar evidences of presentiment almost indefinately, since they are in strict accordance with laws but little understood, and of almost daily occurrance; but this article has already exceeded its alloted space.

